# Grapevine Pathogenic Microorganisms: Understanding Infection Strategies and Host Response Scenarios

**DOI:** 10.3389/fpls.2016.00382

**Published:** 2016-03-30

**Authors:** Grace Armijo, Rudolf Schlechter, Mario Agurto, Daniela Muñoz, Constanza Nuñez, Patricio Arce-Johnson

**Affiliations:** Laboratorio de Biología Molecular y Biotecnología Vegetal, Departamento de Genética Molecular y Microbiología, Facultad de Ciencias Biológicas, Pontificia Universidad Católica de ChileSantiago, Chile

**Keywords:** grapevine, pathogenic microorganisms, infection strategy, host response, resistance and susceptibility

## Abstract

Grapevine (*Vitis vinifera* L.) is one of the most important fruit crop worldwide. Commercial cultivars are greatly affected by a large number of pathogenic microorganisms that cause diseases during pre- and/or post-harvest periods, affecting production, processing and export, along with fruit quality. Among the potential threats, we can find bacteria, fungi, oomycete, or viruses with different life cycles, infection mechanisms and evasion strategies. While plant–pathogen interactions are cycles of resistance and susceptibility, resistance traits from natural resources are selected and may be used for breeding purposes and for a sustainable agriculture. In this context, here we summarize some of the most important diseases affecting *V. vinifera* together with their causal agents. The aim of this work is to bring a comprehensive review of the infection strategies deployed by significant types of pathogens while understanding the host response in both resistance and susceptibility scenarios. New approaches being used to uncover grapevine status during biotic stresses and scientific-based procedures needed to control plant diseases and crop protection are also addressed.

## Introduction

During their lifetime, plants are exposed to a wide variety of pathogens, such as bacteria, viruses, fungi, and nematodes. According to their lifecycle and infection strategies, pathogenic microorganisms can be classified as necrotrophics, biotrophics and hemibiotrophics: necrotrophic pathogens feed on dead tissue, secreting lytic enzymes and phytotoxins to promote cell death into the host plant. Biotrophic pathogens on the other hand, feed on living tissue, developing structures in order to invade the cell and obtain metabolism products. Finally, hemibiotrophic pathogens start with a biotrophic infection phase and then turn to a final necrotrophic phase, killing its host at the end of the infection cycle (Glazebrook, 2005).

Plant defense mechanisms are tightly regulated by hormone-mediated signaling pathways, mainly jasmonic acid (JA) and salicylic acid (SA). It is generally considered that JA and ethylene (JA/Et) mediate necrotrophic pathogens defense, while SA is involved against the biotrophic and hemibiotrophic ones (Pieterse et al., 2009).

Plants respond to necrotrophic pathogens through the induction of JA/Et biosynthesis, which is increased locally and systemically when microorganisms secrete cell wall-degrading lytic enzymes (Robert-Seilaniantz et al., 2011). In addition to wall components, some phospholipids released by the degradation of the plasma membrane directly activate JA biosynthesis (Turner et al., 2002). This increase in JA levels induces the expression of defense related genes coding for glucanases, chitinases, protease inhibitors, and enzymes involved in the biosynthesis of secondary metabolites such as phytoalexins (Glazebrook, 2005).

On the other hand, SA mediates the response to biotrophic and hemibiotrophic pathogens, inducing an increase of reactive oxygen species (ROS) and, therefore, a localized programmed cell death (PCD) in the infected tissue (Pieterse et al., 2009). This defense mechanism, called hypersensitive response (HR), restricts pathogen growth by limiting their access to nutrients and water (Glazebrook, 2005).

For these events to occur, plants must be able to recognize these pathogens. Three types of plant–pathogen interactions have been described to date. The first, PTI or PAMPs-Triggered Immunity (recently called MTI or MAMPs-Triggered Immunity), is a basal immune response activated after recognition of non-adapted pathogens. This corresponds to a first line of defense, common in plants of the same species facing a potentially pathogenic microorganism (Jones and Dangl, 2006; Pieterse et al., 2009). PTI is mediated by plasma membrane localized pattern recognition receptors (PRRs), which are composed of an extracellular domain able to detect PAMPs or MAMPs (i.e., generally structural components of the pathogen) and a intracellular domain that amplifies the signal inside the cell (Monaghan and Zipfel, 2012). A second type of interaction is the effector-triggered susceptibility (ETS), named after the ability of certain microorganisms to overcome the basal plant response through the secretion of virulence factors (effectors) which inhibit PTI, thus promoting the disease (Pieterse et al., 2009). Lastly, there is a third type of interaction known as effector-triggered immunity (ETI). In this case, plants of a particular genotype can identify pathogen effectors through a second class of receptors, called resistance proteins (R). If an effector is recognized by an R protein, either directly or indirectly, it is considered an avirulence factor (AVR) and then the pathogen is avirulent to that plant, since this interaction leads to the activation of HR (Jones and Dangl, 2006; Hayward et al., 2009).

**FIGURE 1:**
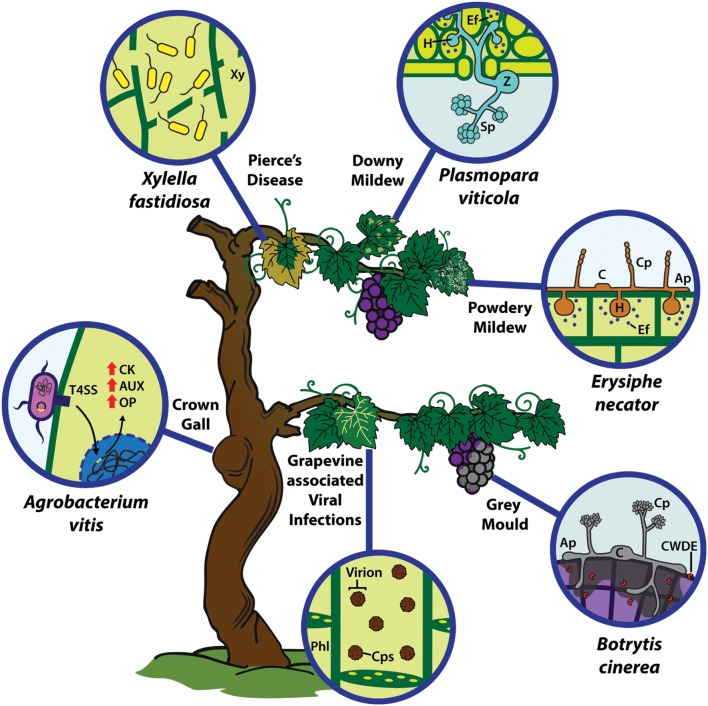
**Infection strategy of main grapevine pathogenic microorganisms and disease-associated symptoms.** Grapevine is affected by different types of microorganisms, i.e., bacteria, fungi, oomycetes and viruses. *Agrobacterium vitis* causes the grapevine crown gall through the injection of T-DNA sequences via a type-IV secretion system (T4SS), incorporating them in the host genome to induce the synthesis of cytokinins (CK), auxins (AUX) and opines (OP). *Xylella fastidiosa* is transmitted by insect vectors; it grows and accumulates within the xylem (Xy) vessels causing Pierce’s Disease. *Plasmopara viticola* zoospores (Z) infect through the stoma in order to accommodate on the host, generating the sporangium (Sp) and generating the grapevine downy mildew (DM) disease. On the other hand, conidia (C) of *E. necator*, the causal agent of the powdery mildew (PM), infects epidermal layers. However, both *P. viticola* and *E. necator* establish their biotrophy by developing haustoria (H) and secreting virulence factors or effectors (Ef) into the host, to manipulate metabolism and defense responses. *Botritys cinerea* conidia (C) germinate and penetrate the plant tissue by necrotizing the host tissue (mainly in grape berries) through the secretion of cell wall degrading enzymes (CWDE), causing the gray mold disease. Viruses are phloem-limited (Phl) microorganisms, whose infections can cause different symptoms in the host. Ap, appresorium; C, conidium; Cp, conidiophore. Cps, capside.

The grapevine (*Vitis vinifera* L.) is one of the most important fruit crops worldwide. This specie is greatly affected by a large number of pathogens that cause diseases in pre- and post-harvest periods, affecting production, processing and export, along with fruit quality. Some of the most important diseases in *V. vinifera* are the gray mold, powdery mildew, and downy mildew (DM), caused by *Botrytis cinerea*, *Erysiphe necator* and *Plasmopara viticola*, respectively, among others (**Figure 1**). The overall purpose of this review is to synthetize the most important grapevine pathogens and their infection strategies along with the host responses in resistance and susceptibility scenarios, taking into consideration the understanding of *Vitis*-pathogens interactions due to its agronomic importance.

## Necrotrophic Pathogens

### Botrytis cinerea

The fungus *B. cinerea* has the ability to live as a parasite in green tissue and as a saprophyte in dead or decaying ones. This is the reason for its wide distribution in nature and host unspecificity. Particularly in *V. vinifera*, *B. cinerea* infection causes a disease known as “gray mold” (**Figure 1**) in post-harvest periods, affecting complete berry clusters during packing, transport and commercialization and thus becoming one of the most important pathogens affecting export wine and table grapes (Dean et al., 2012).

#### Infection Strategy

*Botrytis cinerea* infects grapevine by two main mechanisms: (1) direct mycelium penetration through skin pores or injury, and (2) early invasion, where conidia infect mainly the flower receptacle, and to a lesser extent the stigma and styles, remaining latent within the berry until maturity (Viret et al., 2004). This fungus presents high variability in several biological traits, which can be explained by the great diversity among isolates. However, attachment to a solid surface is a common requisite for conidial germination, though nutritional requirements may differ (Cotoras et al., 2009). When the conidium perceives characteristics and nutrients from the host surface, it develops an infective structure called appressorium that breaches the cuticle by means of a penetration peg (Rolke et al., 2004). It has been described that appressoria-mediated penetration of *B. cinerea* requires a membrane-associated tetraspanin-encoding gene termed *BcPLS1*, since *Bcpls1*-deficient mutants fail to infect intact host cells (Gourgues et al., 2004). The newly formed appressorium secretes lytic enzymes to cross cuticle and outer epithelial wall, like cutinases and lipases, to breach these layers and penetrate the plant cell.

Primary lesion formation triggers an oxidative burst that helps the fungus to kill and degrade the host tissue (Rolke et al., 2004), along with secretion of cell wall degrading enzymes (CWDE) such as endopolygalacturonases (Kars et al., 2005a), pectin methylesterases (Kars et al., 2005b), cellulases and hemicellulases, causing decomposition and consumption of plant biomass. In fact, it has been described that pectin-degrading enzymes are the most abundant plant cell wall modifying proteins expressed during *B. cinerea* infection in grapevines, such as xyloglucan transglucosylase/hydrolases and glucanases (Blanco-Ulate et al., 2014). Pectin breakdown during infection increases the plant cell wall porosity and may facilitate further polysaccharides degradation. Along with this class of enzymes, *B. cinerea* secretes toxins and oxalic acid during infection; the latter acidifies locally the infected region, allowing the activation of pectinases and laccases secreted by the fungus, favoring hyphal growth and inducing morphogenic signaling of infectious structures (van Kan, 2006). Furthermore, oxalic acid contributes to the virulence of the pathogen, promoting both host tissue cell death and sporulation in adjacent cells.

The initial step of infection appears to be similar in berries at different developmental stages, since a recent study of Kelloniemi et al. (2015) revealed that germinated conidia developed appressoria on both veraison and berry maturity, and in the two developmental stages expression of genes related to fungal virulence as endopolygalacturonase (*BcPG2*), pectin methyl esterase (*BcPME2*), superoxide dismutase (*BcSOD1*), glutatione-*S*-transferase (*BcGST1*), among others, were induced during the first 16–24 h. Nevertheless, *B. cinerea* failed to colonize berries at veraison and the infection was only successful in mature berries. In the latter, up-regulated genes were enriched in functional categories related to degradation of plant cell wall (CWDE), proteolysis, membrane transport, ROS generation and detoxification, phytotoxic proteins and secondary metabolites such as botrydial and botcinic acid, which suggest that the initial process of infection and penetration by the fungus may be common in these stages of development, but degradation of plant tissue is a critical step for successful infection.

Physiologically, infection of *B. cinerea* causes significant metabolic changes in berries. High levels of proline, glutamate, arginine, and alanine accumulated in these tissues, accompanied with the production of glycerol, gluconic acid, succinate and largely degraded phenylpropanoids, flavonoid compounds and sucrose (Hong et al., 2012). Moreover, it has been shown that hexokinase activity is required for the development of *B. cinerea* in the presence of hexoses (Rui and Hahn, 2007), suggesting that the fungus is able to manipulate plant metabolism to use carbon sources required for its growth, in addition to products of tissue degradation.

#### Host Response and Resistance against *B. cinerea*

Studies conducted in the *Vitis-B. cinerea* pathosystem showed that grapevines use natural and pre-existing skin qualities as defense structures, such as number of cell layers, cuticle, and wax content of the fruit (Gabler et al., 2003), along with the quantity and density of stomata and leaf trichomes. Also, activation of inducible defense mechanisms during the infection has been reported, such as the expression of polygalacturonase-inhibiting proteins (PGIP) to reduce the extensive pectin degradation caused by fungal attack (De Lorenzo et al., 2001) or an oxidative burst and phytoalexin accumulation in grape cells and primed leaves (i.e., trans-resveratrol and α-viniferin) necessary for defense activation (Aziz et al., 2003; Verhagen et al., 2011). Structural defenses are related to fungal primary infection processes (i.e., appressoria formation and plant tissue penetration), while inducible responses are associated with subsequent infection processes.

Grapevines are also able to secrete a set of chitinases, e.g., *Vvchit1a* (acidic class I chitinase), *Vvchit1b* (basic class I chitinase), and other hydrolytic enzymes to degrade the fungus cell wall (Robert et al., 2002; Chong et al., 2008). However, *B. cinerea* has the capacity to catalyze the conversion of chitin to chitosan, bypassing the degradation of its cell wall (El Gueddari et al., 2002). Thus, a fine-tuning regulation of different layers of plant defense can be deployed to cope at some extend with the pathogen infection.

As described in other plants, necrotrophic-type infection of *V. vinifera* activates the JA/ET pathway along with the induction of genes related to phytoalexin biosynthesis, such as phenylalanine ammonia lyase (*PAL*) and stilbene synthase (*STS*; Chong et al., 2008; Girault et al., 2008; Thaler et al., 2012; Wang et al., 2015). It has been described that methyl jasmonate (MeJA) treatments in harvested grape berries could induce resistance against *B. cinerea* and reduce disease incidence. This is associated with an increase of H_2_O_2_, enhanced expression of the defense-related gene *VvNPR1.1* and accumulation of stilbene phytoalexins, such as tran-resveratrol and its oligomer form, trans-ε-viniferin (Wang et al., 2015). MeJA treatments also induce the biosynthesis of defensive compounds, such as alkaloids, phenolics, flavonoids, and terpenoids along with accumulation of peroxidase, chitinase, β-1,3-glucanase and *PAL* (Jia et al., 2016).

Wan et al. (2015) demonstrated that a minimal production of ROS and a timely elevation of antioxidative capacity were correlated with a high level of resistance in Chinese wild *Vitis*, while highly susceptible cultivar ‘Red Globe’ suffered massive infection and sustained ROS production due to relatively unchanged antioxidative activities, suggesting the importance of ROS response for the timely recognition and defense to *B. cinerea*.

Synthesis and secretion of pathogenesis-related proteins (PR) are also part of the inducible defense mechanism deployed by *V. vinifera*. For instance, up-regulation of *PR-2* in less susceptible plants and accumulation of beta-1,3-glucanases have been described during infection (Derckel et al., 1999). A recent proteomic analysis on *V. vinifera* cell suspension inoculated with *B. cinerea* identified proteins involved in defense (e.g., PR10), response to oxidative stress, cell wall modification and protein folding (Dadakova et al., 2015). Besides, the transcripts of five defensin-like genes (*DEFL*) were identified as significantly up-regulated in grape tissues infected with *B. cinerea* and three novel defensins were proven to inhibit conidia germination (Giacomelli et al., 2012), suggesting a role of these genes in the defense against this pathogen.

A number of transcription factors have also been identified in *V. vinifera* with a significant role in the defense response against *B. cinerea*, whose functional orthologs were previously described in the plant model *Arabidopsis thaliana*. For example, induction of *VvWRKY33* (Group I from WRKY protein family) correlates with the expression of *VvPR10.1*. Complementation of resistance-compromised *Arabidopsis wrky33-1* mutant lines by the constitutive expression of *VvWRKY33* restores the resistance against *B. cinerea* (Merz et al., 2015). Also, transcription factors from the NAC family are known to be involved in the response to environmental stresses, as *VvNAC1* expression is induced in *B. cinerea* infected berries and leaves. Moreover, *VvNAC1*-overexpressing *Arabidopsis* plants exhibit enhanced resistance to this fungus. These plants present a modified expression pattern of defense-associated gene markers (*AtPR-1*, *AtPDF1.2*), suggesting that *VvNAC1* could be a regulatory component of the plant signaling defense cascade (Le Henanff et al., 2013).

The defenses induced in grapevine berries against *B. cinerea* differ during berry ripening. Agudelo-Romero et al. (2015) described the putative involvement of JA/Et, polyamines and auxins, and evidence of a reprogramming of carbohydrate and lipid metabolisms toward increased synthesis of secondary metabolites involved in plant defense, such as trans-resveratrol and gallic acid, and that, contrary to healthy berries, infected green berries did not activate the SA pathway, suggesting that the pathogen is able to shut down some defenses.

On the other hand, inoculated veraison berries accumulate ROS, activate the salicylate-dependent defense pathway, synthesis of the resveratrol phytoalexin, cell wall reinforcement and formation of papillae underneath the appressoria, thus stopping further infection. As was mentioned above, mature grapevine berries are more susceptible than veraison berries. Infected mature berries activate the jasmonate-dependent pathway, which is unable to stop the fungal necrotrophic infection (Kelloniemi et al., 2015).

Grape genotypes vary in their resistance to infection, degree of fungal colonization and severity of disease. Low or no resistant phenotypes in most common table grape *V. vinifera* cultivars have been described, whereas high level of resistance has only been found in the species *Muscadinia rotundifolia* (*V. rotundifolia*), *V. labrusca*, and other grape hybrids. This resistance appears to be related to the number and thickness of epidermal and hypodermal cell layers and cuticle and wax contents (Gabler et al., 2003). However, no genetic studies have been conducted to date in these resistant plants.

To date, pre-existing or basal defenses seem to be an important part of the defense against *B. cinerea*, along with activation of inducible defense mechanisms mediated by SA or JA/Et pathways, depending on the development state, together with an appropriate kinetics between ROS production and generation of antioxidant compounds.

## Biotrophic and Hemibiotrophic Pathogens

### Erysiphe necator

The biotrophic fungus *E. necator* is the etiologic agent of the grapevine powdery mildew (PM; **Figure 1**), affecting species of the genus *Vitis*. This disease appears as a white-grayish powder on the surface of the infected tissue, mostly leaves and stems. This generates large production losses, reduced yields and fruit quality, mainly by a declining in the sugar content and acidity of the berries (Gadoury et al., 2001; Calonnec et al., 2004).

#### Infection Strategy

*Erysiphe necator* depends on its host for growth and development. For this, the conidium attaches to the tissue cells of the plant, allowing the formation of a primary germ tube that differentiates into a specialized infectious structure (i.e., appressorium), which generates a mechanical pressure in order to penetrate and invade the host cell. The successful invasion results in the haustorium formation, by which the fungus absorbs nutrients necessary to complete their life cycle. The haustorium facilitates the dynamic exchange of molecules: the fungus retrieves hexoses, amino acids, vitamins, and other nutrients from host cells, while at the same time secretes proteins to suppress host defenses (Qiu et al., 2015). Once this structure is established, secondary hyphae spread along the infected tissue and finally asexual reproductive bodies (i.e., conidiophores and conidia) emerge from them. When environmental or nutritional conditions become unfavorable, *E. necator* develops cleistothecia, structures of sexual reproduction, that contain four to six asci at maturity, each of which usually contains four ascospores. However, physiological maturity may not be reached for several months, particularly in colder climates. Like conidia, ascospores germinate with a single germ tube, which terminates in appressorium formation (Gadoury et al., 2012).

As in other obligate biotrophic pathogens, *E. necator* genome shows a reduced number of genes related to secondary metabolism (i.e., polyketide synthase, non-ribosomal peptide synthase, dimethylallyl tryptophan synthase and terpene synthase), nitrate and sulfate metabolism, and other core ascomycete genes, such as those related to amino acid metabolism, fermentation, channels/transporters and stress response, among others (Jones et al., 2014). Due to these gene losses, nutrient acquisition from the host is essential for *E. necator* development. Nitrate transporter *VvNPF3.2* has shown to be up regulated in the susceptible grapevine cultivar ‘Cabernet sauvignon’ during PM infection, probably to increase nitrate or nitrite transport, whereas this was not observed in the resistant cultivar ‘Norton’ (Pike et al., 2014). It has also been described in PM-infected *V. vinifera* cv. ‘Cabernet sauvignon’ an increase in the abundance of proteins related to amino acid metabolism (i.e., Alanine aminotransferase and alanine glyoxylate aminotransferases), vitamin biosynthesis and lipid/sterol biosynthesis (Marsh et al., 2010).

In addition, PM exploits as well plant components for its successful penetration and establishment in the host cell. The *MLO*-like class of *R*-genes (Mildew Locus O, MLO, belonging to a family of seven-transmembrane domain proteins) is known to be required for successful host-cell invasion. Loss-of-function *mlo* alleles in barley and *A. thaliana* lead to enhanced resistance to adapted PM species (Freialdenhoven et al., 1996; Consonni et al., 2006). This family is composed by six members in grapevine (*VvMLO3*, *VvMLO4*, *VvMLO6*, *VvMLO9*, *VvMLO13*, and *VvMLO17*), but early induction of *VvMLO3, VvMLO4*, and *VvMLO17* coincided with the establishment of fungal penetration in grape leaves (Feechan et al., 2009). Recently, the locus Sen1 (susceptibility to *E. necator* 1) mapped in *V. vinifera* cv. ‘Chardonnay’ was described as a novel source of PM susceptibility (Barba et al., 2014). Although the biological functions of these genetic components remains elusive, host genes products are likely to be modulated by PM for its successful infection.

#### Host Response and Resistance against *E. necator*

Wine and table grape *V. vinifera* cultivars are very susceptible to *E. necator*. Marsh et al. (2010) showed that susceptible plants (*V. vinifera* cv. ‘Cabernet sauvignon’) are able to initiate a basal defense response but unable to restrict fungal growth or reduce the progression of the disease. Differentially expressed proteins during the infection of *E. necator* are involved in photosynthesis, metabolism, protein destination, and protein synthesis; suggesting that the pathogen manipulates plant energy processes, according to its needs. Furthermore, defense proteins, such as PRs, defensin-like proteins (DEFLs) and proteins involved in ROS detoxification are induced, resembling a basal defense response, however, these mechanisms are inadequate in timing and/or intensity in order to control disease progression (Marsh et al., 2010).

Many species of the *Vitis* genus, mostly from North America, have varying levels of resistance to PM. Feechan et al. (2011) classified this resistance into four types: susceptibility, partial resistance, penetration resistance, and resistance associated with PCD, based on the degree of development of *E. necator* in host tissues and the triggered response. Among these, and as described above, *V. vinifera* is classified as a susceptible species, *V. riparia* as partially resistant and *M. rotundifolia* is among the most resistant species mainly due to the induction of PCD.

At the first level of resistance, *PEN1*- and *PEN2/PEN3*-like pathways [PENETRATION (PEN) genes] are important components of PTI in grapevine, suggesting a role against non-adapted PM. These genes were described in *A. thaliana* and their grapevines orthologs may have similar functions. AtPEN1 is a member of the SNARE (soluble *N*-ethylmaleimide-sensitive factor attachment protein receptor) family which includes proteins that mediate membrane fusion events; AtPEN2 is a myrosinase involved in the biosynthesis of antimicrobial molecules that are delivered to the site of PM penetration via AtPEN3, which is an ATP-binding cassette transporter (Qiu et al., 2015). Their role in PTI response remains elusive, however, it has been shown their importance and functionality in grapevines.

In a second level of resistance, different loci have been identified in several species of the *Vitaceae* family that confer resistance to *E. necator*: REN1 (Hoffmann et al., 2008); REN2 (Dalbó et al., 2001); REN3 (Welter et al., 2007); REN4 (Ramming et al., 2011); REN5 (Blanc et al., 2012); REN6, REN7 (Qiu et al., 2015); RUN1 (Barker et al., 2005); RUN2.1, and RUN2.2 (Riaz et al., 2011), although molecular defense mechanisms triggered by these resistance loci are yet poorly understood. Among them, the RUN1 locus of *M. rotundifolia* was mapped in chromosome 12 and the functional characterization of resistance gene analogs (RGA) within this locus identified a single gene (*RGA10* or *MrRUN1*) that is associated to the complete resistance to PM (Feechan et al., 2013). This gene codes for a resistance protein of the Toll/interleukin-1 receptor-like (TIR)-NB-leucine-rich repeat (LRR) class of immune receptors, composed by a Toll/interleukin-1 receptor-like (TIR), a nucleotide-binding site (NBS) and a leucine-rich repeat (LRR) domains, acting on pathogen effector recognition and signal amplification (McHale et al., 2006). The other resistance loci have also been associated with this type of resistance gene functions; though this has not been experimentally verified to date. Amrine et al. (2015) sequenced and analyzed the transcriptomes of Central Asian grape accessions that were previously shown to carry a REN1-like local haplotype. They confirmed the partial resistance phenotype of these accessions, which is associated with a late post-penetration response to *E. necator* as it was previously described (Hoffmann et al., 2008; Coleman et al., 2009). In all Central Asian accessions, *E. necator* germinated and established primary hyphae, but it failed to colonize extensively the leaf tissues. Necrotic spots that colocalize with appressoria of secondary hyphae suggest that late HR and PCD may be key in slowing down fungal spread in the Central Asian accessions (Amrine et al., 2015).

Effective resistance responses of grapevine against *E. necator* include enhancement of both JA-mediated and systemic acquired resistance (SAR) responses and accumulation of phytoalexins. For example, transcriptomic changes in resistant Chinese wild grapes (*V. pseudoreticulata*) during PM-infection are related to SA and JA responses, SAR, HR, plant–pathogen interaction, flavonoid biosynthesis and plant hormone signal transduction (Weng et al., 2014). Up regulation of defense-related genes such as glycosyl hydrolases, lipases, *PR-5* thaumatin-like proteins and proteinases, among others, has been described in successful response to *E. necator*. For example *VpPR-10.1*, isolated from *V. pseudoreticulata*, has nuclease activity (both RNase and DNase activities) and can lead to PCD and DNA degradation. A correlation between *VpPR-10.1* levels and its antifungal property suggest that the nuclease activity is the biochemical basis for the resistance. Thus, *VpPR-10.1* could potentially play a dual role degrading pathogen RNA and inducing programmed death of host cells (Xu et al., 2014). Also, the transcription factor *VpWRKY1* was rapidly induced in *V. pseudoreticulata* after *E. necator* inoculation, and the expression level was found to be correlated with the resistance magnitude (Li et al., 2010). A number of other genes have also been implicated in PM resistance in certain wild *Vitis* species, showing differential expression levels between PM-resistant wild *Vitis* species and susceptible *V. vinifera* cultivars and conferring increased levels of resistance to PM when overexpressed transiently in grapevine leaves or stably transformed into wild-type or mutant lines of *A. thaliana* (Qiu et al., 2015). The key positive regulator of SA pathway, ENHANCED DISEASE SUSCEPTIBILITY 1 (*EDS1*), was differentially expressed in ‘Norton’ (*V. aestivalis* cv. ‘Norton’) and ‘Cabernet sauvignon,’ where a constitutive expression in the first and an induced in the latter were observed after inoculation with PM (Fung et al., 2008). This also correlates with the high and constitutive SA levels in ‘Norton’ resistant cultivar. Moreover, analysis of the *EDS1* family in grapevine identified both *EDS1* and *EDL2* (EDS1-LIKE 2) in the resistant and susceptible cultivars as necessary components of the regulatory node EDS-PAD4 in a SA-mediated pathway (Gao et al., 2014).

Lignin, flavonoid phytoalexins, and phenolic compounds also play important roles in the defense response of grapes. The rapid production of resveratrol, major compound of the stilbene family and its transformation into viniferins appear to enhance PM resistance in grapevine cultivars. For example, in the resistant cultivar ‘Norton,’ *STS* are induced 24 h after inoculation with PM, accumulate higher levels of *STS* transcripts in mature leaves than the susceptible cultivar ‘Cabernet sauvignon’ and has a differential expression pattern during the berry formation compared to the susceptible cultivar, with a markedly increase at veraison (Dai et al., 2012).

As described above, the presence of resistance loci associated to *R* gene clusters would be crucial to define the susceptibility or resistance of grapevine plants against *E. necator*. This suggests that SA pathway is the main mechanism to lead a successful defense response in *V. vinifera*, since R – AVR signaling is mediated by this hormone. However, other hormones such as JA and the production of secondary metabolites in different cultivars may have a significant role in the defense response against this pathogen.

### Plasmopara viticola

*Plasmopara viticola* (Berk. and Curt.) Berl. and De Toni is the causal agent of grapevine DM (**Figure 1**) and one of the most important pathogens affecting grapevine production worldwide. It was first collected in North East of the USA in 1834. Since then, it has been classified sequentially as the fungus *B. cana*, *B. viticola*, *Peronospora viticola* and finally as the obligate biotrophic oomycete *P. viticola* (Burruano, 2000; Gessler et al., 2011).

*Plasmopara viticola* spread to Europa by the year 1878, probably due to the use of cuttings from American grapes. All cultivated European *V. vinifera* cultivars are susceptible to *P. viticola*. Consequently, the production of *V. vinifera* has been affected by DM to the present. However, several North American *Vitis* species show resistance to this disease at different levels. This characteristic has also been found in *Muscadinia* species and some Asian *Vitis* species. Indeed, the use of natural sources of resistance to pathogens is still a promising tool in breeding programs nowadays (Gessler et al., 2011).

#### Infection Strategy

*Plasmopara viticola* infection occurs specifically through stomata. It starts early in the season, when oospores in fallen leaves or mycelium in dormant twigs are activated by adequate climate conditions to produce sporangia. In presence of water, mature sporangium releases self-motile biflagellate zoospores that infect plants tissues. Zoospores are able to place on the abaxial surface of leaves close to stomata, then germinate and penetrate through the stomatal cavity, where they form a substomatal vesicle. This vesicle gives rise to the primary hyphae and mycelium, which grows through intercellular spaces, enclosed by the veins of the leaf and enters to the cell of the mesophyll by its cell-wall-penetrating and feeding haustoria, which invaginates the plasma membrane of the parenchyma cells. As a result, the adaxial surface of the leaf exhibits a typical oil-spot lesion visible in plants affected by this pathogen. The mycelium also develops to form sporangiophores emerging from the stoma and releasing sporangia to the surrounding susceptible tissues (leaves, twigs, or grape clusters) or plants (Burruano, 2000; Perfect and Green, 2001; Gessler et al., 2011).

Stomata remain abnormally open and unresponsive to abscisic acid in grapevine leaves infected by DM. Recently, Guillier et al. (2015) identified two *V. vinifera* glycoproteins: a phototropin and a lysophospholipase that are induced by pathogen infection, accumulated in apoplastic fluids and considered as candidates for stomatal deregulation.

Little is known about genes involved in pathogenesis in *P. viticola*. During leaf infection, a gene encoding NADH-ubiquinone oxidoreductase has been indicated to be involved in the generation of the proton motive force to allow ATP biosynthesis and active nutrient transport before acquiring them from the host, mainly during the development of the first hyphal structures and haustoria. Analysis of ESTs from germinated zoospores has shown that the most represented unigene encodes a protein similar to fungal laccases, possibly contributing to pathogenesis by playing a role in stilbene detoxification. This could correspond to one of the mechanisms used by the pathogen to counterbalance the production of plant chemical arsenal. Also, an invertase-coding gene would be involved in pathogen proliferation in plant tissues by allowing *P. viticola* to uptake carbohydrates from the host (Mestre et al., 2012; Luis et al., 2013).

In cDNA-AFLP analysis carried out on infected grapevine leaves at oil spot stage, Polesani et al. (2008) identified nine transcripts derived fragments uniquely induced by infection, absent in sporangia, which represent putative virulence factors. In order to identify candidate genes encoding effectors from *P. viticola* several predicted secreted proteins have been identified. Genes corresponding to hydrolytic enzymes, protein inhibitors, elicitor-like proteins and members of the RXLR family of effectors are expressed upon infection. Also, the expression of genes coding for an INL11B-like elicitin, a protein with Kazal-like protease inhibitor fold and a RXLR protein, in infected tissues and germinated zoospores, has been described (Mestre et al., 2012).

#### Host Response and Resistance against *P. viticola*

Yu et al. (2012) established five immunity levels to DM in grapevine: (i) if accumulation of callose deposits around the stomata and inhibition of zoospores germination early in the infection process are observed; (ii) emergence of callose deposits around and in the stomata but unable to inhibit the formation of hyphae; (iii) with callose near the stomata and around haustoria, incapable to stop infection; (iv) with more development of hyphae than in (iii); and (v) with hyphae in all mesophyll tissue intercellular spaces. According to this classification, *M. rotundifolia* was classified as level (i), several Chinese species as level (iii), except for *V. amurensis* as level (iv) and some Chinese species and *V. vinifera* as level (v).

Liu et al. (2015) classified three Chinese wild *Vitis* genotypes according to the histological response to infection. *V. piasezkii* Liuba-8 was classified as ‘highly resistant,’ showing no sporulation, callose deposition and early production of H_2_O_2_, which corresponds to level (i), according to Yu et al. (2012) classification. On the other hand, *V. pseudoreticulata* Baihe-35-1 and *V. davidii* var. cyanocarpa Langao-5 showed decreased levels of sporulation and callose deposits in infected tissues, which could be related to level (ii) in Yu classification (Yu et al., 2012).

In grapevine, some QTLs with major DM-resistance effects have been identified, named Rpv (Resistance to *P. viticola*). Examples of these are Rpv2, derived from *M. rotundifolia* and located on chromosome 18 (Merdinoglu et al., 2003; Peressotti et al., 2010); Rpv3 found in ‘Bianca’ probably from a *V. rupestris* background and mapped on the reference chromosome 18 (Bellin et al., 2009; Casagrande et al., 2011; Di Gaspero et al., 2012); Rpv8, and Rpv12 located on chromosome 14 and Rpv10 located on chromosome 9, from *V. amurensis* (Blasi et al., 2011; Schwander et al., 2012; Venuti et al., 2013).

In the Rpv1 locus derived from *M. rotundifolia* and located on chromosome 12 (Merdinoglu et al., 2003), one of the first grapevine resistance genes to be cloned and functionally characterized was identified, designated *MrRPV1* and conferring strong resistance to *P. viticola* with a broad isolate specificity described by the severe restriction of hyphal growth and sporangiophore development. *MrRPV1* produces four alternatively spliced variants, encoding only one full-length functional TIR-NB-LRR protein (Feechan et al., 2013).

In compatible interactions between grapevine and *P. viticola*, analysis of differentially expressed grapevine genes during infection have demonstrated that most of these genes are down regulated and only 30% up regulated. Genes related to photosynthesis and primary carbon metabolism were the most negatively affected. Other down-regulated genes are related to protein metabolism, transport and signal transduction. Genes involved in secondary metabolism, defense and response to external stimuli were up regulated (Polesani et al., 2008). Among the genes related to defense response, expression of the *PR-2*, *PR-4*, *OSM-1* (osmotin), *GLP-2* (germin-like protein), *GLP-7*, *TLP-4* (thaumatin-like proteins), *CHI* (chalcone isomerase), and *NAC* genes are induced in microdissected stomata and surrounding cells at early times of infection. Thus, there would be a site-specific regulation of grapevine response to *P. viticola*, with short distance signals released from stomata to adjacent cells (Lenzi et al., 2016).

Stress and defense-related genes have shown higher levels of expression in resistant grapevines, so it has been suggested to be linked to constitutive resistance against *P. viticola*. Also, high levels of inositol, caffeic acid, and other antimicrobial substances have been related to constitutive response in uninfected resistant leaves. Up regulation of genes coding for PR proteins occurs as soon as resistant grapevines are inoculated with *P. viticola*. Incompatible interactions are also associated to modulation of genes involved in defense response, photosynthesis, primary and secondary metabolism, signal transduction and transport. This is also followed by an accumulation of metabolites such as resveratrol and viniferins (Gessler et al., 2011; Malacarne et al., 2011).

In *Vitis* progeny analysis carried out by Malacarne et al. (2011), higher stilbenoid accumulation is associated to individuals with least severe DM symptoms. These compounds were trans-resveratrol, trans-piceid, trans-pterostilbene, and several viniferins. Among the genes induced in the incompatible interaction there are two receptor-like protein kinases, one TIR-NBS receptor, a calcium-dependent protein kinase, as well as transcripts concerning ethylene biosynthesis and responsive factors, *PR1*, *PR2*, *PR5*, *PR10*, two isoforms of *STS*, genes encoding a caffeoyl-CoA *O*-methyltransferase, a flavonoid 3′ 5′-hydroxylase and a dihydroflavonol reductase, among others (Malacarne et al., 2011).

The resistance level of grapevines has been correlated to the quantity of the callose deposition. In this context, strong callose deposition in the *P. viticola* resistant *M. rotundifolia* was observed, along with an increased expression of callose synthases genes *CalS1* and *CalS10* at early times of infection (Yu et al., 2012).

*VvWRKY33* has been suggested to play an important role by inducing early defense-related pathways in grapevine leaves against fungal pathogens. VvWRKY33, a class I WRKY transcription factor, activates the promoter of the *VvPR10.1* gene. Both are strongly induced when incompatible interaction occurs; in the case of *VvWRKY33* just 2 h after *P. viticola* infection. Ectopic expression of *VvWRKY33* in grapevine leaves of a susceptible grapevine cultivar has proved to strongly increase resistance to *P. viticola*, reducing sporulation between 50 and 70% (Merz et al., 2015).

Thus, as described in *E. necator*-grapevine pathosystem, some QTLs with major DM-resistance have been identified and would be crucial to define the susceptibility or resistance of grapevine plants against this pathogen. SA pathway, expression of related genes and also production of secondary metabolites may have a significant role in the defense response against this pathogen.

### Agrobacterium vitis

*Agrobacterium vitis* is a specific pathogen of *V. vinifera* and is the causal agent of the grapevine crown gall disease (**Figure 1**). Virulent strains of this bacterium induce the formation of tumorigenic structures at the site of infection for nutrient uptake, while necrosis and a HR-like response has been reported in grapevine roots and in non-host plants infected with this bacterium, respectively. *A. vitis* can be defined as a biotrophic pathogen since it maintains a parasitic relationship with living tissues of their host to complete its life cycle.

#### Infection Strategy

Similar to *Rhizobium radiobacter* (formerly known as *Agrobacterium tumefaciens*), *A. vitis* contains Ti plasmids carrying *vir* genes and T-DNA sequences that allows it to transfer stable genetic material into the genome of its host, particularly genes involved in the biosynthesis of opines, auxins, cytokinins, and the utilization of tartrate (Burr and Otten, 1999). The infection starts commonly through plant injuries, particularly by freezing and/or mechanical wounds, to favor the release of phenolic compounds that act as chemoattractants to the attached bacteria. These compounds activate the transcription of *vir* genes, whose products induce the transfer of the T-DNA into the host genome. VirA is a protein that can detect and interact with the chemical signal and phosphorylate the VirG protein, which in turn transcriptionally activate several other *vir* genes. For instance, VirG activates the expression of *virD2*, whose product has the necessary endonuclease activity to release the T-DNA from the Ti plasmid, and *virE2*, involved in the protection and transport of the bacterial genetic material into the plant (Burr et al., 1998; Burr and Otten, 1999).

The genetic diversity of *A. vitis* is defined by the nature of their Ti plasmids and T-DNA sequences. Ti plasmids are commonly defined according to the type of opine they produce. In grape crown gall, three Ti plasmids have been defined as the octopine/cucumopine (O/C), the nopaline and vitopine plasmids (Szegedi et al., 1988). These three plasmids contain T-DNA sequences that can be categorized into two functional groups, whether are involved in opine synthesis or in gall induction. Opines are low molecular weight compounds derived from aminoacids, keto-acids, and sugar phosphates that only can be catabolized by *Agrobacterium* strains (Escobar and Dandekar, 2003). T-DNAs contain genes for the synthesis of these molecules, such as *acs* (agropine synthase), *cus* (cucumopine synthase), *nos* (nopaline synthase), and/or *vis* (vitopine synthase) depending on the nature of the T-DNA. Moreover, T-DNA sequences also contain genes involved in tumor formation, where genes involved in the biosynthesis of auxins (*iaaM* and *iaaH*) and citokinins (*ipt* genes) can be found (Burr et al., 1998). Oncogenes are also present in these regions, such as the gene *6b* that encodes a protein that stimulates cell growth in an auxin-independent manner. For example, the overexpression of T-6b in transgenic tobacco plants leads to an increased cell expansion in leaf disks but with no apparent changes in auxin content (Clement et al., 2006).

A hallmark of the genetic structure of *A. vitis* is the presence of plasmids with genes encoding proteins involved in tartrate utilization (pTr), an abundant compound in grapevines. Three types of these plasmids have been found in *A. vitis* and were defined as TAR-I and TAR-II from the AB3 strain, and TAR-III from the AB4 strain (Burr and Otten, 1999). Although there are differences in the size of these plasmids, the genes associated with tartrate utilization (*ttuA-E* genes) are conserved among the strains of *A. vitis.* It is suggested that TtuA act as a transcription factor that induce the expression of other *ttu* genes such as *ttuB*, which is involved in the tartrate entry into the bacteria; *ttuC* and *ttuD*, whose product catalyze the degradation of tartrate; and *ttuE*, which codes a tartrate-inducible pyruvate kinase that seems not essential for the utilization of tartrate (Salomone et al., 1996). It is important to notice that the particular characteristic of *A. vitis* to utilize a grape-derived compound can partially explain the high host specificity of this pathogen.

#### Host Response and Resistance against *A. vitis*

Natural resistance sources against the grape crown gall are important for the study of the interaction between tumor-forming pathogenic bacteria and its host, and for the introduction of these sources into further breeding programs. Some examples of both cultivated and wild *Vitis* species are *V. riparia* cv. ‘Glorie de Montpellier’ and *V. amurensis*, respectively, who are resistant to the infection of *A. vitis*. In the case of *V. riparia*, it has been suggested that the T-DNA from *A. vitis* is not stably integrated into the host genome given by the low frequency of T-DNA integration and the absence of opine content in infected tissues (Süle et al., 1994). In *V. amurensis*, a dominant locus has been identified in the chromosome 15 of this specie (*Rcg1* locus), where no signs of crown gall formation were observed on populations carrying this resistant locus (Kuczmog et al., 2012). Even though this resistance seems to affect a wider range of *Agrobacterium* species, the molecular basis underlying the *Rcg1* locus remains unclear.

The Korean cultivar *V. vinifera* cv. ‘Tamnara,’ originated from a grape breeding program between ‘Campbell Early’ (*V. labruscana*) and ‘Himrod Seedless’ (*Vitis* sp.; Park et al., 2004), also showed resistance to the grapevine crown gall. Interestingly, genes related to defense responses in Tamnara were expressed in both inoculations with *A. vitis* and SA-treatments, categorized in groups of ESTs whose genes encode proteins involved in defense (i.e., glucanase, chitinase, thaumatin-like protein, PR-10), signal transduction (NBS-LRR type protein), oxidative burst (i.e., GST, CAT, Glutathione peroxidase) and cell wall fortification (i.e., hydroxyproline-rich protein, extensin-like protein, cinnamyl alcohol dehydrogenase), among others (Choi et al., 2010). It might not be unexpected to find a group of defense related genes that correlate *A. vitis* inoculations and SA treatments in resistant cultivars, since it has been described that SA and SAR have a role in the defense against *R. radiobacter* in *N. benthamiana*, where pre-treatments with SA interferes with the growth and virulence of this bacteria on its host and might affect the expression of several *vir* genes (Anand et al., 2008).

Thus, it seems that the SA signaling pathway may be operating in the interaction between *A. vitis* and resistant cultivars of *V. vinifera*, inducing a defense response similar to those against other biotrophic pathogens mentioned above.

### Xylella fastidiosa

*Xylella fastidiosa* is a gram-negative, xylem-limited bacterium and the causal agent of Pierce’s disease (PD) in *V. vinifera* (**Figure 1**). This bacterium can be classified as a biotrophic pathogen, because it does not kill the host tissue until later stages of its life cycle. The growth of this bacterium depends mostly on climate. The optimum growth conditions for *X. fastidiosa* are warmer environments close to 28°C, this is the main reason that this pathogen is not prevalent in areas where winter temperature drops under 0°C (Lieth et al., 2011).

#### Infection Strategy

*Xylella fastidiosa* is transmitted to new host plants by insect vectors such as sharpshooters (*Homalodisca coagulate*), leafhoppers (*Cicadellidae* family) and spittlebugs (superfamily *Cercopoidea*) during xylem sap feeding. This is the major cause for this pathogen to spread and infect several agriculturally important plants, such as citrus, almond, coffee, and grapevine (Newman et al., 2003). In *V. vinifera*, *X. fastidiosa* causes the Pierce’s disease (PD), an economically important disease that affects wine, table and raisin grape production. The symptoms of this disease can be severe, including leaf scorching, desiccated fruit, cordon die back, and finally vine death (Roper et al., 2007).

*Xylella fastidiosa* infects the vine creating a biofilm in xylem vessels that disrupt water and nutrients flow throughout it. This occlusion is composed by host gums, bacterial exopolysaccharide or degradation products from the host cell wall, or a combination of the three. The xylem is a water transport network of vessels composed of lignified dead cells. The vessels are interconnected by bordered pits (channels) and the pit membranes are composed of pectin, cellulose, hemicellulose and proteins. Pectin polymers determine the pore size in the pit membrane, being sizes between 5 nm in grapevine, allowing the passage of sap through xylem but blocking the passage of larger objects, like *X. fastidiosa*, whose diameter range from 0.3 to 0.5 μm (Davis et al., 1978; Newman et al., 2003; Roper et al., 2007).

Pierce’s disease biofilm formation can be divided in three stages: early biofilm formation, growth, and adaptation. The first stage starts with the transmission of *X. fastidiosa* directly by insect vectors into the xylem vessel. After entry, *X. fastidiosa* needs to move through the vascular system. PilT, a type IV fimbriae, is responsible for retracting and extending fimbriae IV in a twitching motility, allowing the bacteria to move through the vascular system. These processes depend on cell–cell aggregation, which involves membrane attachment proteins, such as chaperonin GroEL, and two-component system regulatory proteins (PopP, FeuP, or PhoP) implicated in the correct adhesion of bacterium-bacterium and biofilm formation (Silva et al., 2011). Following this, the bacteria start to break down xylem pit membranes. In order to do this, they secrete CWDs enzymes, such as endo-1,4-β-glucanases, endoxylanases, β-xylosidases, cellobiohydrolase, and a polygalacturonase (PG). Bacterium needs PG to successfully infect and is a critical virulence factor to promote pathogenesis. This enzyme digests cell wall polymers that form the xylem pit membrane. The digestion of pectin component exposes the cell-wall polysaccharides that are target by the other CWDs enzymes. Thus, the pectin digestion allows *X. fastidiosa* both to use as a carbon source and to move from vessels to vessels (Roper et al., 2007).

The second stage involves a group of metabolic processes to favor biofilm growth, such as amino acid and carbon metabolism and cofactors biosynthesis. Furthermore, at this stage the phenomenon called quorum-sensing (QS) occurs; a bacterial mechanism to sense the population and react to their changing environment. This mechanism is regulated by acylated homoserine lactones (AHLs), which are molecules that trigger the expression of QS regulated genes. QS is involved in the production of *cis*-11-methyl-2-dodecenoic acid, which participates in virulence, motility, toxin production, aerobic respiration, biofilm dispersal and extracellular polysaccharide (EPS). Another important enzyme for this stage is a polynucleotide phosphorylase that degrades plant chloroplasts to provide carbon sources and facilitate the migration through xylem vessels (Roper et al., 2007).

The third and last stage corresponds to adaptation and protection, involving proteins which confer advantages to the bacterial population like resistance to environmental factors. Within this context, toxin production and heat shock protein (sHsp) expression have been observed. sHsp are a small stress induced proteins that prevent aggregation and are important in protein refolding. A predicted membrane localized GTPase was also found, which may be involved in a signal transduction mechanism, but their specific role is to date unclear. Finally, the two-component system regulatory protein (popP, feuP, or phoP) is required for virulence at the last and long stage of infection (Silva et al., 2011).

#### Host Response and Resistance against *Xylella fastidiosa*

The immune response and molecular mechanisms that *V. vinifera* has developed against infection of *X. fastidiosa* remain unclear. However, there are some species, such as *V. labrusca* or *M. rotundifolia* that when infected with *X. fastidiosa*, their symptoms are not as severe as in *V. vinifera* (Lieth et al., 2011). It has been shown that vines employ physical or chemical barriers post infection, such as tylose and gums. Tylose is an outgrowth of parenchyma cells through vessel pit pairs into the lumen of tracheary elements (Sun et al., 2006) and gums are originated from the cell wall or middle lamella (Fry and Milholland, 1990). Some authors suggest that the development of tylose and gums around the infection site is a resistance mechanism. Sun et al. (2006) found that tylose initiation occurred too slowly to restrict spread of mobile pathogens such as *X. fastidiosa*. However, since this bacterium has a slow basipetal movement in systemic infection, tylose could represent a significant obstacle to this type of infection. Fry and Milholland (1990) studied different species of the *Vitis*: *V. vinifera* genus, which is very susceptible to *X. fastidiosa*, and muscadine grapevine (*V. rotundfolia*), whose cultivars are tolerant to PD. The authors demonstrated that the susceptible *V. vinifera* cv. ‘French Colombard’ generates predominantly occlusion pectin, whereas the tolerant *V. rotundfolia* cv. ‘Carlos’ and ‘Noble’ generate occlusion tylose and gums. Thus, the synthesis of tylose and gums could serve as a defense response to contain the infection of *X. fastidiosa*.

Mapping for resistance traits against PD has been reported in hybrids of Mexican cultivars of *V. arizonica*. Within these populations a major QTL has been identified, called PdR1 (Pierce’s disease Resistance 1) in the linkage group 14, which explains 72% of the phenotypic variation (Krivanek et al., 2006; Riaz et al., 2006). Plants carrying this locus have lower levels of bacterial titer in the stems. Moreover, a fine-scale mapping identified two allelic forms in this locus, denominated PdR1a and PdR1b, derived from full siblings of a PD resistant population (*V. rupestris* ‘A. de Serres’ × *V. amazonica/candicans* ‘b43-17’). Within this region, 14 putative genes have been identified and 5 of them were annotated with a molecular function (Riaz et al., 2008). Thus, it is yet to be proved whether PdR1a and PdR1b have different genes within this locus or two alleles of the same gene. A subtractive suppression hybridization (SSH) analysis was performed in leaf, stem, and shoot tissues between resistant and susceptible sibling genotypes (*V. rupestris* × *V. amazonica*) after inoculation with *X. fastidiosa* (Lin et al., 2007). Among these tissues, ESTs derived from resistance genotypes where associated with primary cell wall modifying and metabolic enzymes and PR proteins, such as endo-xyloglucan transglycosylase (EXT), xyloglucan endotransglycosylase (XET), PR-2 (β-1,3-glucanase) and PR-10, among others.

To date, identification of resistance locus to PD, called PdR1, and physical or chemical barriers such as tylose and gums are the most relevant known defense mechanism of grapevine to *X. fastidiosa*.

## Viruses

Nearly 70 virus species have been identified to date that are able to infect the *Vitis* genus, accounting for at least 25 different diseases in grapevine (Martelli, 2014). As other viruses, they are classified by the International Committee on Taxonomy of Viruses (ICTV), classification that is based on several parameters such as: size particle, genome structure (ORFs), nucleotide or amino acid sequence identity of different virus proteins, like heat shock protein 70 (HSP70), polymerases, coat proteins (CP), type of transmission vector and serological information. It should be noted that in many cases they are found associated as a multiplex virus infection complex (Prosser et al., 2007; Martelli, 2014).

From an economic point of view, the most important grapevine viruses are those who cause the leafroll diseases, known as Grapevine Leaf Roll associated Viruses (GLRaV -1, -2, -3, -4, and -7). Moreover, several genetic variants of GLRaV-4 and GLRaV-6 have been described, as well as GLRaV-Pr, GLRaV-Car, and GLRaV-De, based on phylogenetic relationships with the *HSP70h* gene (Maliogka et al., 2009; Ghanem-Sabanadzovic et al., 2010, 2012). All of the above belong to the *Ampelovirus* genus, which harbor a monopartite genome composed of genomic and subgenomics positive (+) single stranded (ss) RNAs with around 12 open reading frames. Two exceptions to the rule have been described: GLRaV-2, classified as a Closterovirus, and GLRaV-7, which has not been assigned to any genus to date (Al Rwahnih et al., 2012a; Naidu et al., 2015).

Other important grape viruses are the Grapevine Virus A and B (GVA and GVB, respectively), belonging to the *Vitivirus* genus, a monopartite genome composed of genomic and subgenomics (+)ssRNAs with five open reading frames (Adams et al., 2004). GVA is related to the Kober stem grooving symptom (Goszczynski et al., 2008; Alabi et al., 2014) and can cause reddening of leaf margins and petioles, poor vigor and leafroll; while GVB is associated with the corky bark syndrome consisting of soft, rubbery, and abnormal swelling of the basal internodes of the canes, longitudinal cracks and cork forming, typical of the rugose wood complex (Bonavia et al., 1996). *Vitivirus* genus has other less ubiquitous species: GVD, GVE, and most the recently discovered GVF, causing similar corky rugose wood-like symptoms (Al Rwahnih et al., 2012b).

On the other hand, the most representative species of grape viruses in the *Nepovirus* genus are the grapevine fanleaf virus (GFLV) and the arabis mosaic virus (ArMV), both causing typical fan-leaf degeneration and leaf decline, reduced fruit quality, short internodes, and abnormal bifurcations. In addition, GFLV is considered to be the major threat to grape industry due to its ability to reduce crop yield up to 80% depending on the isolate, the susceptibility of the grapevine variety and environmental factors (Martelli, 2014).

Not only ssRNA viruses can infect grapes. Al Rwahnih et al. (2013) described a monopartite circular ssDNA virus which belongs to an evolutionarily distinct lineage of the *Geminiviridae* family, the grapevine red blotch-associated virus (GRBaV). This virus causes symptoms such as red blotch, marginal reddening, and red vein in leaves and reduced sugar accumulation in fruits of *V. vinifera* cv. ‘Californian Cabernet Franc,’ ‘Cabernet sauvignon’ and ‘Zinfandel.’ Similarly, a new ssDNA virus from Brazil was described which is able to infect pears, apples and different grapes varieties causing symptoms such as shrinkage, reddening or red blistering of leaves (Basso et al., 2015).

### Infection Strategy

Virus infection starts with the entry of the virus into the plant cell through wounds or graftings, since they cannot cross the cell wall by themselves. In several cases, the spread occurs by transmission vectors, like mealybugs (*Pseudococcidae* family) and dagger nematodes (*Xiphinema index, X. italiae*, and *X. diversicaudatum* among others), which feed on sap phloem or roots, allowing movement of these pathogens into the plant. Also, due to grafting techniques, viruses can flow from an infected bottom (rootstock) to top (scion) through the phloem (Martelli, 2014). Once inside the cell, they must disassemble in order to release their own genome and replicate. Near 70% of plant viruses genomes, including grape-associated ones, are composed of a positive single stranded RNA molecule, or ss(+)RNA. Thus, replication process starts with the expression of a viral replicase, generating negative RNA strands and then subsequently, new positive strands through the activity of a viral RNA dependent RNA polymerase (RdRp; Zabel et al., 1976; Dorssers et al., 1984).

Early translated proteins are involved in the replication process, such as polymerases, helicases, and proteinases. Then, structural proteins such as capsid (CP) and movement proteins (MP) are transcribed, with different functions during viral lifecycle. For example, MP and/or CP are necessary for viral translocation, while CP proteins are also required for virus assembly and cell-to-cell movement (Lucas, 2006). MP proteins can target plasmodesmatas and actively use the host actin/endoplasmic reticulum network to increase the size exclusion limit (SEL) of this structure by either destroying actin filaments and allowing virions to move between cells (Wolf et al., 1989; Waigmann et al., 1994; Harries et al., 2010; Niehl and Heinlein, 2011) or removing desmotubules and expanding membrane pores converting them to tubules (Kawakami et al., 2004; Liu et al., 2005; Schoelz et al., 2011). GFLV replication process occurs in viral compartments from endoplasmic reticulum-derived membranes, and it cannot only can be transported inside Golgi-derived vesicles through microtubule- or microfilament-dependent pathways (Laporte et al., 2003), but they can interact with type-I plasmodesmata located proteins (PDLP) too; these proteins are located on the tubule base of modified plasmodesmatas and genetic disruptions of this interaction can reduce tubule formation, delay infection, and attenuate symptoms (Amari et al., 2010). Nevertheless, not only PDLPs are involved in grape virus movement; class XI myosins (XI-K and XI-2) inactivation produced a mislocalization of PDLP and MP, suppressing GFLV movement, suggesting that these proteins could facilitate the necessary tubule formation for virus translocation (Amari et al., 2011). However, virus translocation is not dependent on virion assembly. This type of cell-to-cell movement can be divided into three steps: first, genome transfer from replication sites to intracellular transport systems; then, assisted intracellular transport of the vira1 genome and finally, the intercellular transport of genomes through plasmodesmata-TMV-like mechanisms (Carrington et al., 1996).

Finally, in phloem-restricted viruses (**Figure 1**), particles must translocate throughout the plant tissues, moving from mesophyll cells to sieve elements and thus achieve a systemic infection. Viruses take advantage of the phloem vasculature and sink-to-source flux in order to passively move through the plant at a very fast rate (Leisner and Turgeon, 1993). Although many viral determinants have been proposed depending on the viral genus, CP and MP seem to play a major role in this long-distance movement (Hipper et al., 2013). In the end, they are released into systemic tissues, thus starting new infection sites.

### Host Response and Resistance against Viral Infection

The common plant cell response to viral infection is the activation of HR, including cell wall fortification and induction of PCD to block the systemic propagation of the virus (Stange et al., 2008; Komatsu et al., 2010). This response has been associated to an R gene-mediated immunity (i.e., ETI), through recognition of AVRs by this class of host immune receptors (Jones and Dangl, 2006). Nevertheless, this kind of response has not been reported within *Vitis* genus and no direct resistance gene has been described (Martelli, 2014).

During compatible grapevine–virus interactions, host transcriptional reprogramming in both leaves and berries has been reported. For example, presence of GRLaV-3 in *V. vinifera* cv. ‘Carmenere’ was correlated with leaf red coloration and chlorosis. Transcriptomic and developmental changes were studied during fruit ripening in *V. vinifera* cv. ‘Cabernet sauvignon’ infected with GRLaV-3. Reduction in sugar and total anthocyanins content, followed by differentially expressed genes, were observed during grape–virus compatible interaction. Among repressed genes, those associated with sugar metabolism, transport, and the phenylpropanoid pathway were severely inhibited at the ripening stage (E-L38), while genes associated to cell rescue, defense, death and aging were up-regulated (Vega et al., 2011). Similar results were found by Espinoza et al. (2007a,b) were same cellular responses plus proteasome-ubiquitin pathway and other biological processes as transport, transcription and RNA processing were induced during viral infections. Moreover, in *V. vinifera* cv. ‘Merlot’ infected with GRLaV-3, there is an induction of genes related to the flavonoid biosynthetic pathway, specifically the anthocyanin branch, which contributes with the reddish-purple leaf coloration phenotype in GRLaV-3-infected grapevines (Gutha et al., 2010). The same author showed that accumulation of anthocyanins, flavonols and proanthocyanidins are correlated with the induction of chalcone synthase (CHS), flavonoid-3′-hydroxylase (F3′H), flavonoid-3′, 5′-hydroxylase (F3′5′H), dihydroflavonol reductase (DFR), leucoanthocyanidin dioxygenase (LDOX), UDP-glucose:flavonoid 3-*O*-glycosyltransferase (UFGT) and the transcription factor MYBA1.

A physiological, agronomical and transcriptomic analysis of the infection of *V. vinifera* cv. ‘Bosco’ by GRSPaV (Grapevine rupestris stem pitting associated-virus) has been conducted by Gambino et al. (2012). At veraison (E-L35), infected grape leaves showed a decrease in their chlorophyll content and net photosynthesis, although up-regulation in photosynthesis, hormones, and secondary metabolism-related genes was observed. However, defense, signal transduction and primary metabolism (TCA, glycolysis and pentose phosphate pathway) related genes were down regulated in this infected tissue. In the same study, GRSPaV-infected berries at veraison showed that 233 genes were differentially expressed, showing down regulation of main functional categories (i.e., stress, disease, senescence, ROS detoxification, ethylene and JA signaling). It is believed that GRSPaV and grapevine have co-existed for a long period of time and these changes account as a form of adaptation and co-evolution between these two species, particularly in the inactivation of stress and defense responses and the favoring of the induction of photosynthesis associated genes.

Infected hosts are able to trigger molecular changes during compatible interactions in order to restrain virus infections. Plants deploy two main endogenous mechanisms to regulate gene expression: transcriptional gene silencing (TGS), which involves decreased RNA synthesis because of promoter methylation (Meyer et al., 1993; Sijen et al., 2001) and post-transcriptional gene silencing (PTGS; de Carvalho et al., 1992; Van Blokland et al., 1994) that recognizes dsRNA as a target (Hammond et al., 2001), which are then trimmed by type-III RNAses (i.e., DICER), producing small RNA fragments (i.e., siRNA). These short fragments act as a signal for a complementary sequence RNA that can be degraded at the end of the cycle. TGS and PTGS were considered as separate pathways. TGS was thought to regulate mainly transposons and accidental transgenes that mimic transposons. By contrast, PTGS was thought to regulate viral infection and accidental transgenes that encode some types of aberrant RNA that mimic viral RNA. The major breakthrough in the distinction came with the discovery that viruses and transgenes encoding dsRNA induce either TGS or PTGS (Vaucheret and Fagard, 2001).

As such, host PTGS machinery might be able to recognize viral dsRNA as a surveillance mechanism to cope with viral infections. For this reason, efforts have been made in order to obtain viral induced silencing transgenic lines expressing sense, antisense or both viral sequences in grapevines. For instance, *V. rupestris* and 110 Richter rootstock were successfully transformed with a short CP sequence using embryogenic cultures (Krastanova et al., 1995). Another transformations involved sense and antisense-oriented CP gene sequences from GFLV and GLRaV-3, a truncated HSP90-related gene of GLRaV-3 in rootstocks Couderc 3309, *V. riparia*, Teleki 5C, Millardet et De Grasset 101-14, and 110 Richter (Xue et al., 1999), and the GVA MP sense or antisense sequence expressed in *V. rupestris* plantlets (Martinelli et al., 2002).

However, virus can counterdefense plant response against infections in at least two known ways: RNAi viral suppressor proteins (VSRs) and RNA silencing suppressors (RSSs; Li and Ding, 2006; Ding and Voinnet, 2007; Murray et al., 2013). In either case, viruses can block the RNA silencing pathway at different stages (Burgyan and Havelda, 2011), affecting dsRNA processing and silencing signal amplification, decreasing siRNA stabilization, suppressing RISC activity or acting as suppressors with unspecified functions (Alvarado and Scholthof, 2009). For example, GLRaV-2 p21 protein suppresses PTGS through direct binding to siRNAs (Ye and Patel, 2005). Also, GLRaV-2 p24 protein and GVA p10 protein have been described to possess silencing suppressor activity (Chiba et al., 2006; Zhou et al., 2006). Another way to counterdefense plant response are protein domains encoded by RNA viruses which decrease alkylation damage. Van den Born et al. (2008) demonstrated that an Alk-B domain encoded by five viruses from the Flexiviridae family, is able to demethylate RNA with a robust repair activity due to its N-terminal extensions beyond the Alk-B core. Several other enzymes are also involved in viral RNAi suppression. Proteinase1/Helper component-proteinase (P1/HC-Pro) acts as a suppressor of both transgene induced and virus-induced gene silencing in tobacco plants (Anandalakshmi et al., 1998). Also, a tandem GLRaV-2 papain-like leader proteases, L1 and L2, are essential to establish the infection process of GLRaV-2 in initially inoculated grapevine (Liu et al., 2009).

Because viruses can affect plant post-transcriptional gene silencing mechanisms, some authors worked on a way to attack this viral counterdefense response. Soler et al. (2012) found that when three VSRs: p25, p20, and p23 from citrus tristeza virus (CTV) are silenced, complete resistance to viral infection in some transgenic lines were obtained. Thus, this strategy could be a promising approach to induce virus resistance in grapevine.

To date, identification of resistance genes has not been reported within *Vitis* genus, however, virus defense on plants is a common mechanism involving different steps on regulatory pathways. Future research for a deeper understanding of these mechanisms in grapes and other species is required.

## Disease Management Outlook

Disease diagnosis is a time-consuming laborious task, especially regarding the identification of a particular pathogen and selection of the best control strategy to prevent or reduce economic losses. However, concerning grapevine disease management, some strategies available are very expensive and/or not tolerated by the importing markets, mainly due to their potentially negative environmental impact.

Since the vast majority of *V. vinifera* cultivars are susceptible to the pathogens abovementioned in this review, wine and fresh-grape production depends greatly on the use of effective control methods. Cultural control techniques, including thinning and pruning, “cold curing” (i.e., exposing plants to low temperatures to prevent or eliminate the pathogen), chemical control including contact and systemic compounds, biological control and natural products have been included in management systems. Also, in some grapevine disease models applied to decision support systems, other variables have been correlated with the pathogen life cycle, as meteorological conditions and phenological stage of the plant to reduce the use of pesticides applications and increase management efficiency (Gessler et al., 2011; Lieth et al., 2011; Kamoun et al., 2015).

Among the most effective methods, chemical control is one of the most used strategies. Based on the type of chemical (active ingredient), “pesticides” can be classified as copper compounds, sulfur compounds, dithiocarbamates, benzimidazoles, antibiotics, among others. Although these chemicals are effective against fungal and bacterial pathogens, they are not effective against viral diseases. Despite this, certain highly volatile chemicals compounds are toxic for organisms including insects, common viral vectors. Nevertheless, this technique is not highly effective and cannot avoid future infections. For this reason, management control programs nowadays use healthy mother plants insuring virus-free next generation plants (Almeida et al., 2013). Another strategy is to use silencing sequences of suppressor viral proteins that have the potential to avoid future viral infections. For example, the first attempts to artificially induce RNA silencing pathways in grapevines has been done in transgenic *V. vinifera* cv. “Chardonnay” expressing artificial miRNA targeting GFLV CP (Jelly et al., 2012).

Currently, main concerns in disease management include the environmental and health impact of chemical compounds. Biological control of grapevine and plant diseases in general has become more significant, especially against fungi as *B. cinerea*, by using antagonistic microorganisms before or after infection (Calvo-Garrido et al., 2014; Parafati et al., 2015). However, the use of disease-resistant cultivars of *V. vinifera* is the most cost-effective, safe, and environmentally desirable option, since resistant cultivars sometimes offer the only practical control option available. The identification and inclusion of natural sources of resistance in grapevine breeding programs by pyramiding two or more resistance genes or loci, looking for a sustainable viticulture in the future is one of the most commonly used practices. Some of these plants are used as rootstocks, since some plants have substantial levels of resistance, but are not of commercial interest. However, a few *Vitis* species have been found to be resistant to these pathogens, nevertheless, the source of this resistance has not been completely identified.

The use of biotechnology, for example in the generation of genetically modified plants carrying these resistance elements, could be an effective strategy accepted in the future in agriculture. New molecular approaches are being developed, suggesting that the editing of *cis*-regulatory elements (CRE) in defense related genes could be a new target in order to increase pathogen resistance (Swinnen et al., 2016). All the aforementioned are excellent candidates to use with novel genome editing tools, such as CRISPR/Cas9being introduced for crop plants with promising results. Big data approaches are also being develop for plant-pathogen interactions in order to facilitate information accessibility for crop developers. Examples as the PhytoPath (Pedro et al., 2016), which provides access to all plant pathogen genomes submitted to the International Nucleotide Sequence Database Nucleotide Consortium are of great significance. It would be an advantage to include this type of approaches for the development of resistant grape cultivars with minimal genome modifications.

At present, integrated disease management, using combined methods to eliminate or reduce pathogen threats and negative impact on the environment and health is a partially effective, economical, and sustainable way to cope with these problems.

## Concluding Remarks

Understanding plant responses to different kind of pathogens may show some light on the co-evolution of plant and pathogen strategies, and their impact on resistance or susceptibility to infections.

The study of plant–pathogen interactions in crop plants, like grapevine, is essential to understand how pathogens infect the plant and how plant defenses are activated and enhanced. The understanding of these processes allows looking for biotechnological applications to reduce the economic losses associated to these microorganisms. It is crucial to reduce the handling and treatment with chemical agents, but it is necessary to go deeply into the molecular processes that underlie these interactions in order to achieve this.

## Author Contributions

GA, RS, MA, DM, CN, and PA-J designed the work; made the work drafting, did data collection and reviewed it critically for the final version. RS designed the figure.

## Conflict of Interest Statement

The authors declare that the research was conducted in the absence of any commercial or financial relationships that could be construed as a potential conflict of interest.
